# The Effect of Excipients on the Permeability of BCS Class III Compounds and Implications for Biowaivers

**DOI:** 10.1007/s11095-015-1773-4

**Published:** 2015-08-19

**Authors:** Alan Parr, Ismael J. Hidalgo, Chris Bode, William Brown, Mehran Yazdanian, Mario A. Gonzalez, Kazuko Sagawa, Kevin Miller, Wenlei Jiang, Erika S. Stippler

**Affiliations:** GlaxoSmithKline Inc., Research Triangle Park, North Carolina 27709 USA; Absorption Systems LP, Exton, Pennsylvania 19341-2556 USA; US Pharmacopeial Convention, Rockville, Maryland 20852 USA; Teva Branded Pharmaceuticals R&D Inc., West Chester, Pennsylvania 19380 USA; P’Kinetics International, Inc., Pembroke Pines, Florida 33027 USA; Pfizer Global Research and Development, Groton, Connecticut 06340 USA; Food and Drug Administration, Office of Generic Drugs, Silver Spring, Maryland 20841 USA

**Keywords:** BCS class III, bioavailability, Caco-2, permeability, rat intestinal perfusion model

## Abstract

**Purpose:**

Currently, the FDA allows biowaivers for Class I (high solubility and high permeability) and Class III (high solubility and low permeability) compounds of the Biopharmaceutics Classification System (BCS). Scientific evidence should be provided to support biowaivers for BCS Class I and Class III (high solubility and low permeability) compounds.

**Methods:**

Data on the effects of excipients on drug permeability are needed to demonstrate that commonly used excipients do not affect the permeability of BCS Class III compounds, which would support the application of biowaivers to Class III compounds. This study was designed to generate such data by assessing the permeability of four BCS Class III compounds and one Class I compound in the presence and absence of five commonly used excipients.

**Results:**

The permeability of each of the compounds was assessed, at three to five concentrations, with each excipient in two different models: Caco-2 cell monolayers, and *in situ* rat intestinal perfusion. No substantial increases in the permeability of any of the compounds were observed in the presence of any of the tested excipients in either of the models, with the exception of disruption of Caco-2 cell monolayer integrity by sodium lauryl sulfate at 0.1 mg/ml and higher.

**Conclusion:**

The results suggest that the absorption of these four BCS Class III compounds would not be greatly affected by the tested excipients. This may have implications in supporting biowaivers for BCS Class III compounds in general.

## Introduction

The Biopharmaceutics Classification System (BCS) is a framework for classifying drug substances based upon their aqueous solubility and permeability across biological membranes (1). The FDA BCS guidance (2) provides recommendations for sponsors of investigational new drug applications (INDs), new drug applications (NDAs), abbreviated new drug applications (ANDAs), and supplements to these applications (SNDA) who wish to request a waiver of *in vivo* bioequivalence (BE) studies for immediate-release (IR) solid oral dosage forms. Drug substances are classified based on their intestinal permeability (or the fraction of oral dose absorbed) and aqueous solubility at multiple pH values covering the range found in the gastrointestinal (GI) tract. The solubility class boundary is based according to Papadopoulou *et al.* (3) the highest dose strength of the drug substance in a pharmaceutical product that is dissolved in 250 ml (8 oz.) of aqueous buffer.

Class I drugs have high permeability, and high solubility at all pH values between 1.0 and 7.5 (original FDA guidance from 2000), between 1.0 and 6.8 (new FDA draft guidance, May 2015) (2) or between 1.2 and 6.8 (European Medicines Agency (EMA) and World Health Organization (WHO) (4,5); such compounds are well absorbed but may have poor bioavailability (BA) due to extensive first-pass metabolism. Class II compounds have high permeability, but their solubility is below the class boundary at one or more pH values (e.g., at low pH for acids or at neutral pH for bases). The fraction absorbed may be limited by their solubility; therefore, it is not uncommon to see a wide range of extent of absorption for this class of compounds. Class III drugs have low permeability and high solubility, and their fraction absorbed is sometimes limited by their permeability. Class IV drugs have both low permeability and low solubility.

Class I drugs can be granted waivers from *in vivo* BE testing (“biowaivers”) due to their consistently high fraction absorbed, regardless of formulation (6). Several authors have suggested that Class III compounds should also be eligible for biowaivers if a new formulation does not change the permeability or the GI transit time of the drug (7–9). WHO recommends, and the new FDA draft guidance (2) makes provision for, biowaivers for BCS Class III drug products, but only in cases where the generic and comparator drug products are very rapidly dissolving and the excipients meet certain criteria (e.g., qualitatively the same, quantitatively very similar, well-established for use in products containing that drug substance, do not affect GI motility or interactions with transport processes, and do not affect the pharmacokinetics (PK) of the drug substance) (2,5). This suggestion has also been proposed based upon a theoretical assessment of drug BA (10,11).

Certain excipients have been shown either to enhance the *in vitro* permeability of drugs (e.g., by changing membrane integrity or affecting transporters) or to modify GI transit time (12,13). Limited human data also suggest that some excipients may alter the BA of BCS Class III drugs (14,15). Several of these excipients have been demonstrated to alter membrane permeability directly, but others may work by inhibiting secretory transport mechanisms (16).

In order to determine if a new formulation of a BCS III drug is suitable for a biowaiver, the potential effect(s) of the excipient(s) on permeability need to be evaluated. If no effect can be discerned in a well-validated *in vitro* model, it is reasonable to expect no change in permeability *in vivo* as well (17–21). First described for this purpose in 1989 (17), the utility of the Caco-2 cell monolayer model for qualitative (e.g., rank-order or high *vs.* low) prediction of the oral absorption of drugs has been validated repeatedly since then (18–21).

This series of experiments had three objectives: 1) to determine whether a series of commonly used excipients would alter the permeability of model drug compounds in both a Caco-2 cell monolayer system (*in vitro* model) and an *in situ* rat intestinal perfusion model (surrogate for human *in vivo* data); 2) to determine whether there was a correlation between the Caco-2 and *in situ* rat intestinal perfusion models; and 3) to evaluate the relative suitability of the two models for testing the effects of excipients. The excipients were selected on the basis of their aqueous solubility and prevalence in IR formulations.

Five excipients were selected for testing: hydroxypropyl methylcellulose (HPMC), povidone, polyethylene glycol (PEG)-400, sodium lauryl sulfate (SLS), and lactose. All of these are commonly used in oral formulations, and some have been shown to act as penetration enhancers (16). The model compounds evaluated for permeability were antipyrine (BCS Class I), acyclovir (Class III), atenolol (Class III), ganciclovir (Class III), and nadolol (Class III) (22). The Class III compounds were selected because they are not substrates of P-gp and because they represent a range of the *in vivo* fraction absorbed, with atenolol > nadolol > acyclovir > ganciclovir (23). Antipyrine was included as a highly permeable control compound.

## Materials and Methods

### Materials

Ganciclovir was obtained from Toronto Research Chemicals (Toronto, Ontario, Canada). Acyclovir, antipyrine, atenolol, nadolol, digoxin, propranolol, d-glucose, d-lactose monohydrate, povidone K15, HPMC, PEG-400, SLS, 2-(*N*-morpholino)ethanesulfonic acid (MES), and Krebs Ringers buffer (KRB) were obtained from Sigma-Aldrich (St. Louis, MO, USA). Lucifer yellow (LY), Hanks’ balanced salt solution (HBSS), Dulbecco’s modified Eagle’s medium (DMEM), and 4-(2-hydroxyethyl)-1-piperazineethanesulfonic acid (HEPES) were obtained from Invitrogen (Carlsbad, CA, USA). Fetal bovine serum (FBS) was obtained from Omega (Tarzana, CA, USA). Penicillin, streptomycin, non-essential amino acids (NEAA), and trypsin-EDTA were obtained from CelGro (Herndon, VA, USA). Costar^®^ tissue culture flasks and dual-chamber Transwell^®^ plates were obtained from Corning (Corning, NY, USA).

The excipients used and their respective concentrations are given in Table I. The selection of the excipients and the concentrations used in the study were based on information obtained from the FDA’s Inactive Ingredient Database (24). The inclusion criteria for selection of the excipient and the concentrations included the frequency at which the excipient is used (they are all commonly used), the amount of excipient typically used, the solubility of the excipient in physiological fluids, and the concentration of the excipient in the commonly used dosing volume of 250 ml. This may be an important factor in determining the choice of excipients in new formulations.

**Table I:** Concentrations of Excipients Tested in the Two Permeability Models

Excipient	Concentrations In Caco-2 (mg/ml)	Concentrations in rat intestinal perfusion (mg/ml)
Lactose	0.024, 0.13, 0.24, 1.0, 2.0	0.024, 0.13, 0.24
HPMC	0.012, 0.036, 0.06, 1.0, 2.0	0.012, 0.036, 0.06
PEG-400	0.015, 0.06, 0.18, 0.30	0.06, 0.18, 0.30
Povidone	0.024, 0.042, 0.06, 0.1, 0.2	0.024, 0.042, 0.06
SLS	0.01, 0.02, 0.04, 0.10, 0.17	0.01, 0.02, 0.04, 0.17

### Bioanalysis

A liquid chromatography–tandem mass spectroscopy (LC-MS/MS) analytical method was developed and validated for the simultaneous determination of the five compounds (acyclovir, antipyrine, atenolol, ganciclovir, and nadolol) in the presence of five excipients (HPMC, povidone, PEG-400, SLS, and lactose). The analytical system was a PE SCIEX API 3000 LC-MS/MS with Flux Instruments Rheos 2000 pumps, a CTC Analytics autosampler, and an on-line degassing system. The LC column was a Thermo Aquasil C18 (3 μm, 50 × 2.1 mm) run at 300 μl/min with a 9-min gradient from 90% water/10% ammonium formate buffer (40 mM, pH 3.5) to 90% acetonitrile/10% ammonium formate buffer. Accuracy, based on analysis of replicate standards at 1 μM, was between 98.8 and 107% for all analytes, with coefficients of variation ranging from 2.6 to 5.9%. Similar accuracy and precision were found at lower concentrations.

### Caco-2 Cells

Caco-2 cells (clone C2BBe1) were cultured in DMEM supplemented with 10% FBS, 1 mM sodium pyruvate, 100 μM NEAA, 2 mM L-glutamine, 100 U/ml penicillin, and 100 μg/ml streptomycin. Stock cultures were grown in 175-cm^2^ flasks at 37°C in a humidified atmosphere containing 5% CO_2_. Cells were harvested with trypsin-EDTA, seeded at a density of 60,000 cells/cm^2^ on collagen-coated Transwell plates (0.4-μm pore size), and grown at 37°C in a humidified atmosphere containing 5% CO_2_. The culture medium was changed every other day for 10 days, and daily thereafter. Cell monolayers on Transwell plates were used for transport assays between 21 and 28 days post- seeding. Prior to use, each batch of monolayers was certified by measuring the transepithelial electrical resistance and the permeability of atenolol, propranolol, LY, and digoxin.

### Permeability Across Caco-2 Cell Monolayers

#### Non-specific Binding and Recovery Assessment

Acyclovir (100 μM), antipyrine (10 μM), atenolol (100 μM), ganciclovir (100 μM), and nadolol (100 μM) were dosed together as a cassette in the apical chamber of a cell-free Transwell device (*n* = 3 replicates) to determine the non-specific binding (recovery) and cell-free apparent permeability across the membrane of each compound. Samples were collected from the donor and receiver chambers at multiple time points and analyzed by LC-MS/MS. “Cassette” refers to a small set of drugs (e.g., 3–5) or other compounds that are dosed together (co-dosed) for evaluation of their permeability (*in vitro*), absorption (*in situ*), or BA (*in vivo*). The assumption is that they do not interact with each other or interfere with each other’s absorption, in which case the co-dosed results should reflect the results that would be obtained if they were dosed individually.

#### Permeability Assay

The unidirectional (apical-to-basolateral (A→B)) and bidirectional (A→B and B→A) permeability of acyclovir, antipyrine, atenolol, ganciclovir, and nadolol, dosed as a cassette, was determined with Caco-2 cell monolayers. Bidirectional permeability was measured in the absence of excipients only (*n* = 3 replicates), whereas unidirectional permeability was measured both in the presence and absence of excipients (*n* = 4 replicates). The transport buffer was HBSS, supplemented with 10 mM MES or HEPES buffer and 10 mM D-glucose (15 mM D-glucose final concentration). The pH of the buffer in the apical (A→B donor, B→A receiver) chamber was 6.8, and the pH of the buffer in the basolateral (A→B receiver, B→A donor) chamber was 7.4. The *apparent permeability (P*_app_) and recovery were calculated as follows:

$$ \begin{array}{l}{P}_{\mathrm{app}} = \left(d{C}_r/dt\right)\times {V}_r/\left(A\times {C}_0\right)\hfill \\ {}\mathrm{Percent}\ \mathrm{Recovery} = 100 \times \left[\left({V}_{\mathrm{r}}\times {C_{\mathrm{r}}}_{\mathrm{final}}\right) + \left({V}_{\mathrm{d}}\times {C}_{\mathrm{d}\ \mathrm{final}}\right)\right]/\left({V}_{\mathrm{d}}\times {C}_0\right)\hfill \\ {}\mathrm{Effluxratio}={P_{\mathrm{app}}}_{\left(B\to A\right)}/{P_{\mathrm{app}}}_{\left(A\to B\right)}\hfill \end{array} $$

*dC*_r_/*dt* is the rate of appearance of the compound in the receiver compartment (μM/sec); *V*_r_ and *V*_d_ are the volumes of the receiver and donor compartments, respectively (cm^3^); *A* is the surface area of the cell monolayer (1.13 cm^2^); *C*_0_ is the concentration (μM) of the dosing solution; and *C*_r final_ and *C*_d final_ are the concentrations (μM) in the receiver and donor compartments, respectively, at the end of the incubation period.

### Permeability Across Rat Jejunum in Recirculating Intestinal Perfusion Model

#### Test System

Sprague–Dawley rats (300–350 g) were used to perform closed-loop, *in situ* intestinal perfusion. Rats were fasted for 12–18 h before the experiment, and water was supplied *ad libitum*.

#### Study Design

Four rats (*n* = 4) were used per treatment group, with a total of 17 groups: one control group (assay buffer, no excipients) and three or four treatment groups for each of the five excipients, with a different concentration for each group. The recirculating intestinal perfusion was performed with the five compounds dosed as a cassette (100 μM acyclovir, 10 μM antipyrine, 100 μM atenolol, 100 μM ganciclovir, and 100 μM nadolol) in KRB with 10 mM MES, pH 6.8.

### Test Method

Rats were anesthetized with an intraperitoneal injection of ketamine/xylazine (1 ml/kg) and placed on a heating pad to maintain normal body temperature. After making a midline incision, the intestines were externalized. A 10-cm segment of the jejunum was tied off, cannulated at both ends to form a loop, and flushed with saline and air. The loop was equilibrated statically with dosing solution for 30 min, then flushed with air and perfused by recirculation (peristaltic pump) with 10 ml dosing solution at 0.2 ml/min. Samples of the perfusate were taken from the reservoir at 15, 30, 45, 60, 75, and 90 min for analysis by LC-MS/MS.

#### Sample and Data Analysis

The effective permeability coefficient, P_eff_ (cm/s), was calculated as

$$ {P}_{eff} = \hbox{-} \left(d\left( ln\left[C/{C}_0\right]\right)/dt\right)\times V/2\pi rl $$

where *C* is the concentration at a given time and *C*_*0*_ is the concentration in the dosing solution (μM); *d(*ln *C/C*_*0*_*)/dt* is the slope of a plot of the natural log of the concentration ratio in the perfusate *vs.* time (sec^−1^) at steady state, corrected for the apparent loss (due to dilution) of the impermeant marker FD-4 (fluorescein-conjugated dextran with a molecular weight of 4 kDa); *V* is the volume of the dosing solution (10 cm^3^); 2π*rl* is the luminal surface area (cm^2^), based on a radius (*r*) of 0.178 cm for the rat intestine (25) and the measured length of each intestinal segment, *l* (cm).

## Results

### LC-MS/MS

The LC-MS/MS method for acyclovir, antipyrine, atenolol, ganciclovir, and nadolol cassette analysis—in the presence and absence of the excipients HPMC, povidone, PEG-400, SLS, and lactose—had adequate sensitivity and selectivity and was valid for use in this study. The lower limit of quantification (LLOQ) was 0.005 μM, and the upper limit of quantification (ULOQ) was 1 μM for all analytes. The intra- and inter-assay accuracy and precision were within ±12% of nominal at all concentration levels in all three matrices (HBSSg, pH 7.4; HBSSg, pH 6.8; and KRB, pH 6.8). Benchtop, autosampler, and refrigerator stability were adequate, and no significant endogenous interference was found from HPMC, povidone, SLS, PEG-400, or lactose.

### Bidirectional Permeability

As shown in Table II, all five compounds in the cassette had recoveries of at least 85% and high cell-free permeability under A→B test conditions. High recovery indicates little loss to either non-specific binding to the device or chemical instability during the assay, and high cell-free *P*_app_indicates that the compounds were freely diffusible across the membrane (lack of aggregation, etc.).

**Table II:** Non-specific Binding and Recovery of Antipyrine, Acyclovir, Atenolol, Ganciclovir and Nadolol from Cell-Free Permeability Test Devices

Compound (μM)	Cell-free P_app_ (10^−6^ cm/s)^a^	Recovery (%)
Acyclovir (100)	29.8 ± 1.8	86.1 ± 1.7
Antipyrine (10)	34.8 ± 0.8	85.8 ± 2.1
Atenolol (100)	33.8 ± 0.7	102 ± 1.1
Ganciclovir (100)	31.1 ± 1.4	92.3 ± 1.6
Nadolol (100)	33.0 ± 0.7	101 ± 1.5

Data presented as mean ± SD, *n* = 3

^a^ For example, a value of “1.0” represents a P_app_ of 1.0 × 10^−6^ cm/s

The bidirectional permeability results presented in Table III demonstrate that the BCS Class I compound antipyrine had much higher permeability across Caco-2 cell monolayers than the other four model compounds in the cassette (all of which were BCS Class III compounds), with complete recovery and no significant efflux (efflux ratio was approximately equal to 1). The permeability of acyclovir, atenolol, ganciclovir, and nadolol were each quite low and similar in magnitude to each other; the efflux ratios ranged from 2.1 to 2.7.

**Table III:** Bidirectional Permeability and Recovery of Antipyrine, Acyclovir, Atenolol, Ganciclovir and Nadolol Across Caco-2 Cell Monolayers

Compound (μM)	A→B		B→A		Efflux ratio^b^
	P_app_ (10^−6^ cm/s)^a^	Recovery (%)	P_app_ (10^−6^ cm/s)^a^	Recovery (%)	
Acyclovir (100)	0.32 ± 0.14	82.2 ± 8.6	0.85 ± 0.11	94.9 ± 12.2	2.7
Antipyrine (10)	44.5 ± 0.70	104 ± 3.1	52.5 ± 3.80	98.8 ± 9.60	1.2
Atenolol (100)	0.29 ± 0.14	83.6 ± 9.2	0.62 ± 0.08	94.0 ± 11.0	2.2
Ganciclovir (100)	0.32 ± 0.16	82.9 ± 7.3	0.70 ± 0.09	92.7 ± 10.8	2.1
Nadolol (100)	0.29 ± 0.16	83.9 ± 9.1	0.71 ± 0.15	90.7 ± 12.2	2.5

Data presented as mean ± SD, *n* = 3

^a^ For example, a value of “1.0” represents a P_app_ of 1.0 × 10^−6^ cm/s

^b^ Efflux ratio is calculated from the mean P_app_ value in each direction

### Unidirectional Permeability

The concentrations of the excipients tested are shown in Table I. The concentrations (in mg/ml) are shown with the two permeability models, Caco-2 cells and rat intestinal perfusion.

The results presented in Table IV demonstrate no consistent, concentration-dependent changes in the permeability of the model compounds across Caco-2 cell monolayers in the A→B (physiological absorptive) direction in the presence of up to 2.0 mg/ml lactose, 0.2 mg/ml povidone, 2.0 mg/ml HPMC, 0.04 mg/ml SLS, or 0.3 mg/ml PEG-400. The same was true for monolayer integrity (data not shown). On the other hand, SLS at concentrations of 0.1 and 0.17 mg/ml increased the post-experiment permeability of the monolayer integrity marker LY, indicative of damage to the cell monolayer (see footnote to Table IV). The permeability of the model compounds in the presence of 0.1 mg/ml SLS was approximately 10-fold higher than control; permeability in the presence of 0.17 mg/ml SLS was not determined due to the evident cell monolayer damage.

**Table IV:** Effects of Excipients on *In Vitro* Caco-2 Cell Monolayer Permeability

Excipient	Conc., mg/ml	Apparent permeability coefficient (P_app_) (10^−6^ cm/s)				
		Antipyrine	Acyclovir	Atenolol	Ganciclovir	Nadolol
Control^a^		39.9 ± 2.14	0.28 ± 0.06	0.23 ± 0.05	0.25 ± 0.06	0.21 ± 0.06
Control^b^		43.6 ± 0.89	0.28 ± 0.06	0.25 ± 0.05	0.22 ± 0.05	0.21 ± 0.04
D-Lactose	0.024^a^	42.3 ± 5.53	0.18 ± 0.04	0.19 ± 0.05	0.14 ± 0.04	0.14 ± 0.04
	0.13^a^	41.6 ± 0.58	0.32 ± 0.11	0.57 ± 0.46	0.48 ± 0.42	0.44 ± 0.37
	0.24^a^	39.8 ± 0.55	0.31 ± 0.20	0.27 ± 0.19	0.28 ± 0.19	0.27 ± 0.20
	1.0^b^	33.3 ± 0.85	0.20 ± 0.05	0.16 ± 0.06	0.17 ± 0.05	0.14 ± 0.05
	2.0^b^	33.1 ± 1.32	0.17 ± 0.14	0.14 ± 0.01	0.16 ± 0.01	0.13 ± 0.01
Povidone	0.024^a^	44.5 ± 0.91	0.17 ± 0.02	0.14 ± 0.01	0.15 ± 0.01	0.11 ± 0.01
	0.042^a^	40.4 ± 0.96	0.21 ± 0.03	0.15 ± 0.02	0.18 ± 0.03	0.13 ± 0.02
	0.06^a^	41.9 ± 0.30	0.14 ± 0.004	0.11 ± 0.003	0.12 ± 0.002	0.09 ± 0.02
	0.1^b^	38.5 ± 1.56	0.19 ± 0.05	0.13 ± 0.03	0.16 ± 0.04	0.12 ± 0.03
	0.2^b^	36.3 ± 0.56	0.23 ± 0.06	0.16 ± 0.05	0.19 ± 0.05	0.15 ± 0.05
HPMC	0.012^a^	40.8 ± 0.99	0.15 ± 0.01	0.12 ± 0.01	0.14 ± 0.01	0.11 ± 0.01
	0.036^a^	37.3 ± 1.33	0.18 ± 0.04	0.14 ± 0.04	0.15 ± 0.04	0.14 ± 0.03
	0.06^a^	39.2 ± 1.63	0.17 ± 0.05	0.14 ± 0.05	0.15 ± 0.05	0.14 ± 0.05
	1.0^b^	33.2 ± 1.51	0.20 ± 0.06	0.15 ± 0.05	0.16 ± 0.05	0.13 ± 0.05
	2.0^b^	33.5 ± 0.29	0.17 ± 0.03	0.13 ± 0.02	0.15 ± 0.03	0.11 ± 0.02
SLS	0.01^a^	46.9 ± 2.50	0.25 ± 0.07	0.24 ± 0.07	0.21 ± 0.07	0.23 ± 0.07
	0.02^a^	40.9 ± 1.11	0.34 ± 0.04	0.32 ± 0.05	0.30 ± 0.04	0.31 ± 0.05
	0.04^a^	45.9 ± 0.56	0.35 ± 0.11	0.39 ± 0.12	0.30 ± 0.10	0.31 ± 0.10
	0.1^a,c^	36.6 ± 1.23	2.48 ± 1.08	2.14 ± 0.96	2.23 ± 0.98	1.98 ± 0.87
	0.17	Not determined due to disruption of monolayer integrity				
PEG 400	0.015^b^	40.7 ± 0.96	0.34 ± 0.04	0.29 ± 0.04	0.30 ± 0.04	0.27 ± 0.04
	0.06^a^	38.9 ± 0.49	0.17 ± 0.01	0.17 ± 0.01	0.16 ± 0.01	0.13 ± 0.01
	0.18^a^	37.2 ± 0.53	0.22 ± 0.06	0.18 ± 0.05	0.20 ± 0.05	0.14 ± 0.04
	0.30^a^	37.2 ± 0.68	0.19 ± 0.06	0.16 ± 0.05	0.18 ± 0.05	0.12 ± 0.04

Data presented as mean ± SEM (*n* = 4)

^a,b^ Two separate sets of controls were run, each in parallel with a batch of test wells with excipients

^c^ Integrity of Caco-2 cell monolayers was compromised (LY P_app_ 0.18 × 10^−6^ cm/s with buffer only; 0.30 × 10^−6^ cm/s with test compounds only (no excipients); 0.05 × 10^−6^ cm/s with 0.01 mg/ml SLS; 0.16 × 10^−6^ cm/s with 0.02 mg/ml SLS; 0.20 × 10^−6^ cm/s with 0.04 mg/ml SLS; 2.20 × 10^−6^ cm/s with 0.1 mg/ml SLS; 4.61 × 10^−6^ cm/s with 0.17 mg/ml SLS)

### Permeability in the Recirculating *In Situ* Rat Intestinal Perfusion Model

**Fig. 1:**
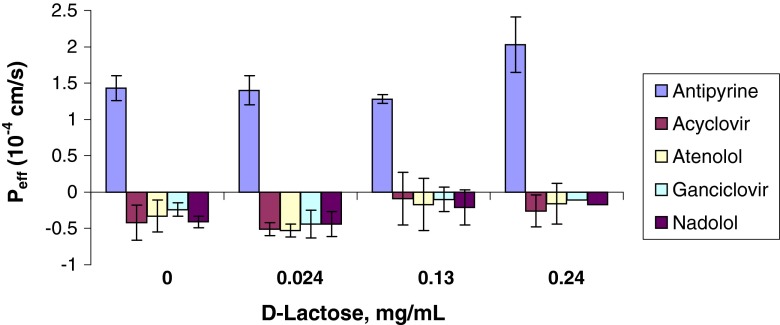
P_app_ *vs.* d-Lactose concentration.

**Fig. 2:**
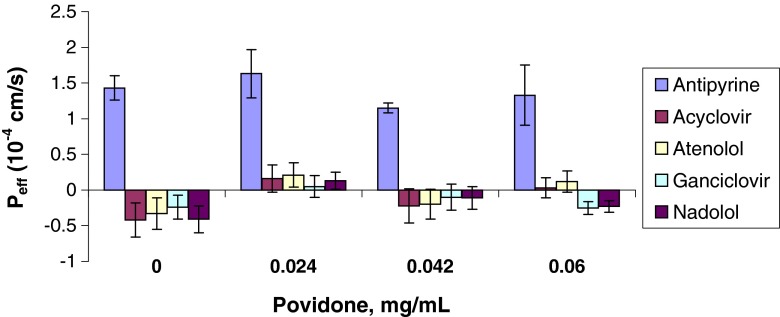
P_app_ *vs.* Povidone concentration.

**Fig. 3:**
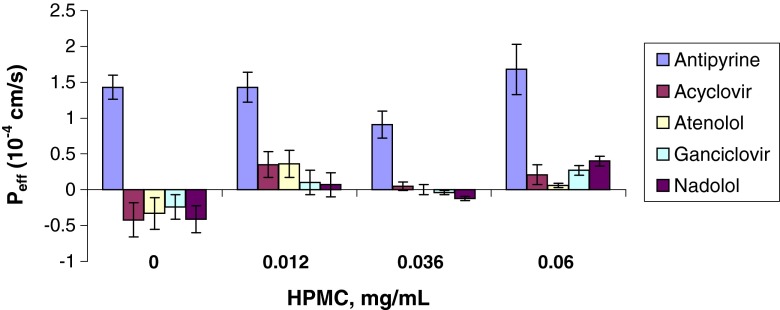
P_app_ *vs.* HPMC concentration.

**Fig. 4:**
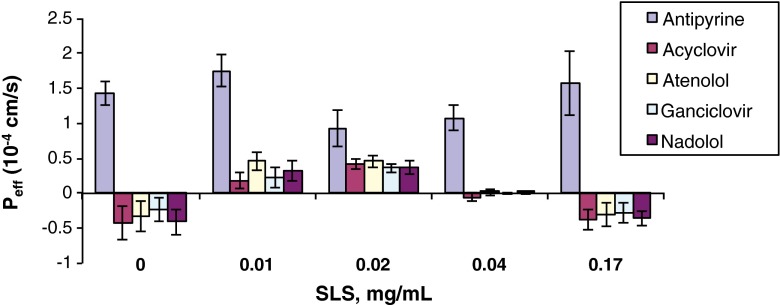
P_app_ *vs.* SLS concentration.

**Fig. 5:**
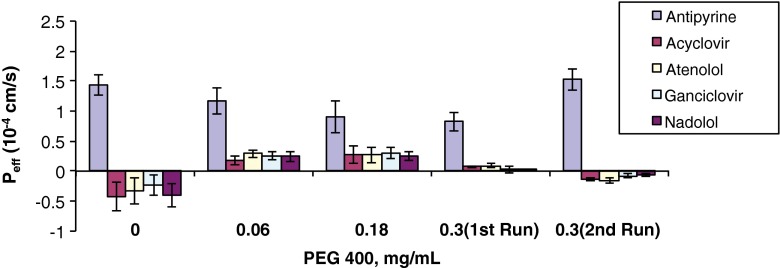
P_app_ *vs.* PEG-400 concentration.

As shown in Table V and in Figs. 1, 2, 3, 4, and 5, the permeability results in the recirculating *in situ* rat intestinal perfusion model also suggest that lactose (up to 0.24 mg/ml), povidone (up to 0.06 mg/ml), HPMC (up to 0.06 mg/ml), SLS (up to 0.17 mg/ml), and of PEG-400 (up to 0.3 mg/ml) do not have a substantial effect on the permeability of the model compounds across rat jejunum. The small sample size precludes definitive conclusions regarding statistical significance.

**Table V:** Effects of Excipients on *In Situ* Rat Jejunal Permeability

Excipient	Conc., mg/ml	Effective permeability coefficient (P_eff_) (10^−4^ cm/s)				
		Antipyrine	Acyclovir	Atenolol	Ganciclovir	Nadolol
Control		1.43 ± 0.17	−0.42 ± 0.24	−0.33 ± 0.22	−0.24 ± 0.17	−0.41 ± 0.19
D-Lactose	0.024	1.40 ± 0.20	−0.51 ± 0.09	−0.53 ± 0.09	−0.44 ± 0.09	−0.44 ± 0.08
	0.13	1.28 ± 0.06	−0.09 ± 0.36	−0.17 ± 0.36	−0.10 ± 0.19	−0.21 ± 0.17
	0.24	2.03 ± 0.38	−0.26 ± 0.22	−0.16 ± 0.28	−0.11 ± 0.17	−0.17 ± 0.24
Povidone	0.024	1.63 ± 0.34	0.16 ± 0.19	0.21 ± 0.17	0.05 ± 0.15	0.13 ± 0.015
	0.042	1.15 ± 0.07	−0.22 ± 0.24	−0.20 ± 0.21	−0.10 ± 0.18	−0.11 ± 0.16
	0.06	1.33 ± 0.42	0.03 ± 0.14	0.12 ± 0.15	−0.25 ± 0.09	−0.23 ± 0.08
HPMC	0.012	1.43 ± 0.21	0.35 ± 0.18	0.36 ± 0.19	0.10 ± 0.17	0.07 ± 0.17
	0.036	0.91 ± 0.19	0.05 ± 0.06	0.00 ± 0.07	−0.04 ± 0.03	−0.12 ± 0.03
	0.06	1.68 ± 0.35	0.21 ± 0.14	0.06 ± 0.03	0.27 ± 0.07	0.40 ± 0.07
SLS	0.01	1.75 ± 0.23	0.19 ± 0.012	0.46 ± 0.13	0.23 ± 0.15	0.32 ± 0.15
	0.02	0.93 ± 0.26	0.41 ± 0.07	0.46 ± 0.09	0.36 ± 0.07	0.37 ± 0.10
	0.04	1.08 ± 0.18	−0.05 ± 0.07	0.02 ± 0.05	0.00 ± 0.01	0.01 ± 0.02
	0.17	1.57 ± 0.46	−0.38 ± 0.14	−0.30 ± 0.17	−0.28 ± 0.14	−0.36 ± 0.10
PEG 400	0.06	1.17 ± 0.22	0.17 ± 0.07	0.29 ± 0.07	0.26 ± 0.07	0.24 ± 0.08
	0.18	0.90 ± 0.26	0.27 ± 0.14	0.27 ± 0.13	0.30 ± 0.09	0.25 ± 0.08
	0.30	0.82 ± 0.15	0.07 ± 0.02	0.09 ± 0.03	0.02 ± 0.05	0.01 ± 0.01
	0.30^a^	1.53 ± 0.18	−0.21 ± 0.19	−0.13 ± 0.20	−0.24 ± 0.13	−0.23 ± 0.13

Data presented as mean ± SEM (*n* = 4)

^a^Second run

The permeability data were noticeably less variable in the Caco-2 model than in the *in situ* rat perfusion model: median relative standard deviation for antipyrine of 2.64% in the Caco-2 model *vs.* 18.5% in the rat perfusion model; a range of 17.0 to 19.4% for the Class III compounds in the Caco-2 model *vs.* 46.4 to 65.2% in the rat perfusion model.

## Discussion

An LC-MS/MS method was validated for acyclovir, antipyrine, atenolol, ganciclovir, and nadolol determinations as a cassette in the absence and presence of excipients. This approach allowed for a relatively rapid assessment of the results of both arms of the study. This approach may be used to test the effects of other excipients on the same model compounds.

The permeability of all five model compounds across intestinal epithelia was measured both *in vitro* (Caco-2 cell monolayers) and *in situ* (rat intestinal perfusion). One problem with the intestinal perfusion model is that negative P_eff_ values are often observed for low-permeability compounds. The most likely reason for a negative P_eff_ value is that net water flux cannot be assessed accurately. When a small amount of a supposedly “impermeable” marker (FD-4 in this case, used to correct for water secretion) is absorbed, mathematically it results in overcorrection for water secretion, resulting in negative P_eff_ values for low-permeability compounds. Currently, there are no perfect markers for the rat intestinal perfusion model. FD-4 was used as the impermeable marker in this study, since this marker is widely accepted in the literature. Although negative P_eff_ values for low-permeability compounds suggest that the rat perfusion model may be variable and potentially insensitive to small changes in drug absorption, this system is widely accepted for the evaluation of permeability.

The permeability of the BCS Class I compound antipyrine across Caco-2 cell monolayers was high in both directions, with no asymmetry (ratio of B→A *P*_app_ to A→B *P*_app_ near unity). The Caco-2 A→B (absorptive direction) permeability of the Class III model compounds acyclovir, atenolol, ganciclovir, and nadolol were all very low and nearly identical in magnitude (between 0.29 × 10^−6^ and 0.32 × 10^−6^ cm/s). The efflux ratios of the Class III compounds ranged from 2.1 to 2.7, a moderate degree of apparent efflux that is due in part to the fact that, in each case, the recovery was somewhat lower in the A→B direction than in the B→A direction, which would tend to underestimate the *P*_app_ in the A→B direction (the denominator in the efflux ratio), leading to an overestimate of efflux. Clinically, linear peak plasma concentration as a function of oral dose (PK) has been reported for atenolol (26) and nadolol (27), suggesting no significant interaction with efflux transporters. For acyclovir (28) and ganciclovir (29), decreasing exposure as a function of dose has been reported after oral administration; this is evidence for possible involvement of active intestinal uptake and is not consistent with an interaction with efflux transporters.

With the exception of an effect of SLS on Caco-2 cell monolayers at concentrations of 0.1 and 0.17 mg/ml, where cell monolayer integrity was clearly compromised, none of the tested excipients caused a substantial increase in the permeability of any of the model compounds in either model. The effect of SLS on Caco-2 cell monolayer integrity at higher test concentrations was clearly a model-specific toxic effect, and therefore may not be pharmaceutically relevant. Other studies have reported positive effects of excipients on the permeability of different drugs, but the effects appear to be dependent on the excipient and its concentration (12,30–32). For example, sorbitol and SLS reduce the bioavailability of the Class I drug risperidone, the former due to increased GI motility and the latter by an unknown mechanism (33). With the exception of SLS at the two highest tested concentrations, the excipients used in this study do not appear to disrupt cell monolayer integrity at the concentrations used, whereas some of the excipients used in other studies (e.g., sodium caprate (31)) may be more likely to do so.

This study focused on the effects of individual excipients on permeability and did not evaluate the effects of combining multiple excipients (which would be commonly done in an IR formulation) on permeability. Because of the vast number of excipient combinations and concentration combinations, testing for the synergistic effects of combinations was not feasible.

While the limitations of the Caco-2 cell monolayer model (like any other preclinical model) are appreciated by investigators in the field, the FDA does accept Caco-2 data for BCS classification. In spite of its limitations, based on the fact that Caco-2 permeability data have lower variability between replicates than data obtained from *in situ* recirculating rat intestinal perfusion, the Caco-2 cell monolayer model may be more sensitive in terms of detecting effects of an excipient on drug permeability. On the other hand, Caco-2 cell monolayers may be overly sensitive to some excipients, as with SLS at or above 0.1 mg/ml. Therefore, a positive result may need to be followed up *in vivo* to rule out a false positive.

Except for SLS at concentrations at or above 0.1 mg/ml (at which concentrations the effects are on monolayer integrity rather than due to permeability enhancement), the results of this study with a set of four BCS Class III model compounds support the more general hypothesis that these five commonly used excipients do not affect the permeability of BCS Class III compounds. Biowaivers for Class III compounds should be considered when excipients used in a formulation have been shown not to affect permeability. There are many recent examples of biowaivers granted by the WHO for Class III drugs (34–38), as well as the assessment that BE of many of the IR drug products available today can be assured with an *in vitro* dissolution test (39). The results of this study support biowaivers for BCS Class III compounds. This would reduce the amount of time needed to get new formulations to patients who need them, reduce the cost of developing new formulations, and reduce the cost of modifying or improving the manufacturing process for products containing Class III drugs. From an ethical point of view, it also reduces the needless exposure of healthy volunteers to drugs without therapeutic benefit.

## Conclusion

The influence of five excipients (lactose, povidone, HPMC, SLS, and PEG400), commonly used in IR formulations, on the permeability of five model drugs (acyclovir, antipyrine, atenolol, ganciclovir, and nadolol) was investigated. Two test systems were used: Caco-2 cell monolayers and *in situ* recirculating rat intestinal perfusion; both have been used for absorption enhancement studies. Within the tested concentration range for all drug–excipient combinations, the excipients caused no substantial increases in drug permeability, except in the case of SLS at concentrations (0.1 mg/ml and higher) where cell monolayer integrity was compromised. These results should support biowaivers for BCS Class III (high solubility–low permeability) compounds, but only for compounds similar in permeability to those tested and which have been formulated with the excipients used in this study.

## ABBREVIATIONS

BA: Bioavailability
BCS: Biopharmaceutics classification system
BE: Bioequivalence
IR: Immediate-release
KRB: Krebs Ringers buffer
LC-MS/MS: Liquid chromatography-tandem mass spectroscopy
LY: Lucifer yellow
MES: 2-(*N*-morpholino)ethanesulfonic acid
NEAA: Non-essential amino acids
P_app_: Apparent permeability coefficient
PEG: Polyethylene glycol
PK: Pharmacokinetics
SD: Standard deviation
SEM: Standard error of the mean
